# Prognostic Value of Growth Differentiation Factor 15 in Kidney Donors and Recipients

**DOI:** 10.3390/jcm9051333

**Published:** 2020-05-03

**Authors:** Ulrich Jehn, Katharina Schütte-Nütgen, Ute Henke, Joachim Bautz, Hermann Pavenstädt, Barbara Suwelack, Stefan Reuter

**Affiliations:** Department of Medicine D, Division of General Internal Medicine, Nephrology and Rheumatology, University Hospital of Münster, 48149 Münster, Germany; ulrich.jehn@ukmuenster.de (U.J.); katharina.schuette-nuetgen@ukmuenster.de (K.S.-N.); henke.ute@gmx.net (U.H.); joachim.bautz@ukmuenster.de (J.B.); hermann.pavenstaedt@ukmuenster.de (H.P.); barbara.suwelack@ukmuenster.de (B.S.)

**Keywords:** kidney transplantation, renal disease, growth differentiation factor-15, GDF15, living donation, dialysis

## Abstract

Growth differentiation factor-15 (GDF15) is associated with inflammatory conditions, chronic kidney disease, cardiovascular disease and mortality. There is very limited data on GDF15 after kidney donation and transplantation. We analyzed serum samples of patients who donated a kidney (54 living donors) or who underwent kidney transplantation (104 recipients) at the University Hospital of Münster (Germany) between 2013 and 2015, for GDF15 levels immediately prior and one year after surgery. GDF15 levels were significantly elevated in end-stage renal disease patients compared to healthy individuals (2844 (IQR 2087, 3361) pg/ml vs. 384 (IQR 307, 487) pg/ml, *p* < 0.001). GDF15 was strongly associated with the dialysis vintage. While kidney transplantation led to a significant decrease of GDF15 (913 (IQR 674, 1453) pg/ml, *p* < 0.001), kidney donation caused a moderate increase of GDF15 (510 (IQR 420, 626), *p* < 0.001) one year after surgery. GDF15 levels remained significantly higher in recipients and kidney donors than in healthy controls (735 (IQR 536, 1202) pg/ml vs. 384 (IQR 307, 487) pg/ml, *p* < 0.001). GDF15 is increased in patients with kidney disease and is associated with dialysis vintage. Given its decrease after transplantation and its increase after uni-nephrectomy, GDF15 might be a marker of kidney function. However, since it correlates only to the eGFR in transplanted patients it may indicate chronic kidney disease.

## 1. Introduction

Growth differentiation factor-15 (GDF15) or macrophage inhibitory cytokine-1 (MIC-1), belongs to the superfamily of transforming growth factor β (TGF-β) widely expressed in mammalian tissues [1]. Since increased GDF15 levels are strongly associated with cardiovascular disease and all-cause mortality [2], it is not surprising that GDF15 was postulated to be an independent serum marker of mortality in chronic kidney disease (CKD) and hemodialysis patients [3,4,5].

Its expression is highly regulated and GDF15 is often induced in response to conditions associated with inflammation, oxidative stress, and hypoxia, among others [6]. Depending on the condition of the cells and the microenvironment, GDF15 can have both protective and adverse effects [7]. For example, Luan et al. demonstrated in mice that GDF15 was necessary for surviving both bacterial and viral infections, including sepsis. Since the observed effects of GDF15 were largely independent of the pathogen control or the extent of the inflammatory response, they suspected a role for GDF15 in disease tolerance as e.g. GDF15 was required for hepatic sympathetic outflow and triglyceride metabolism [8]. In diabetic mice, genetic deletion of GDF15 led to increased damage in the tubules and interstitium but not in glomeruli, hinting towards a tissue-protective effect of GDF15 in kidneys [9]. Interestingly, the kidney itself expresses GDF15 in response to different injuries including ischemic and toxic damage [10,11].

In humans, there are few studies linking GDF15 to kidney disease. Nair et al. found circulating GDF15 levels to be correlated with GDF15 expression in the tubulo-interstitial compartment and with the progression of CKD. Since it was found to be independent of other risk factors, the authors postulate that GDF15 is not only a risk factor but rather involved in the progression of CKD [12]. 

After kidney transplantation (KTx), Connelly et al. observed a significant decrease in GDF15 levels in their patients within twelve months. They found an inverse correlation between GDF15 and eGFR after KTx [13]. The same correlation was found by Malysko et al. who additionally showed that GDF15 levels were still increased in kidney allograft recipients when compared to healthy volunteers [14]. The same was true for children [15]. 

Since only one longitudinal GDF15 study reporting data of 37 patients within one year after KTx and no study on GDF15 after kidney donation (KD) has been published, our study, which analyzes 4-year data adds substantial insights into the role of GDF15 after KTx and KD, respectively.

## 2. Experimental Section

### 2.1. GDF15

Plasma concentration of GDF15 was measured by Human GDF15 DuoSet ELISA (R&D Systems, Minneapolis, MN, USA) according to manufacturer’s instructions. The assay range is 7.81–500 pg/mL, with an unspecified sensitivity for GDF15. Samples above the concentration limit for the test were re-measured after 10-fold dilution in Calibrator Diluent RD6-10 reagent according to manufacturer’s specifications.

### 2.2. Study Population

We prospectively included 104 consented patients (age ≥ 18 years) who underwent kidney transplantation at our transplant center between April 2013 and October 2015. Patients were followed until February, 2019. Fifty-four living kidney donors (LD) served as controls.

The GDF15 was measured in a time frame of 24 hours prior and one year after KTx or KD, respectively. After KTx, the initial immunosuppressive regimen consisted of tacrolimus (target trough 6–12 ng/mL), mycophenolate mofetil, and prednisolone. The transplant recipients received induction therapy with basiliximab; anti-thymocyte globulin was administered to those with a high degree of immunization (PRA > 85%) or a previous transplantation. ABO-incompatible patients received rituximab four weeks prior to KTx. Two patients with atypical hemolytic uremic syndrome as underlying disease received eculizumab in combination with basiliximab. Oral CMV-prophylaxis with valganciclovir was administered for 100 days in R+ and for 200 in the D+/R− constellation.

The characteristics of the patients and the LD were taken from the electronic patient file. Data of all patients were anonymized before the analysis. Written informed consent was obtained from all participants. All experiments on humans were performed in accordance with the current transplantation guidelines and the Declarations of Istanbul and Helsinki. This study was approved by the local ethics committee (Ethik Kommission der Ärztekammer Westfalen-Lippe und der Medizinischen Fakultät der Westfälischen Wilhelms-Universität, No. 2013-364-f-S and No. 2019-109-f-S).

### 2.3. Outcome Measures

The main outcome measures were renal function (eGFR calculated by CKD-EPI equation and urine-protein/creatinine ratio (UPCR)) in years one to four after surgery. Further outcome parameters were patient and overall graft survival. Patient survival was defined as the time from KTx until death (regardless of cause) or last contact for living patients. Overall graft survival was defined as the time from KTx until death (from any cause), graft failure, or last contact, whichever occurred first. Graft failure was defined as the re-initiation of dialysis or re-transplantation. Since too few patients reached the endpoints graft loss or death within our observation period, we analyzed the combined endpoint death, graft failure or eGFR loss > 30% as primary endpoint.

Kidney biopsy was performed if creatinine levels increased (≥0.3 mg/dL) and/or a significant proteinuria occurred. The kidney biopsies were evaluated by two pathologists. Rejections were diagnosed by histological biopsy evaluation based on the BANFF classification [16]. Whole blood was analyzed for creatinine (enzymatic assay; Creatinine-Pap, Roche Diagnostics, Mannheim, Germany). Proteinuria was assessed using spot urine. CMV-DNAemia was assessed as published previously [17]. In short, viremia was considered relevant if >214.6 copies/mL corresponding to the threshold value given by the manufacturer (90% CI 163 to 355 IU/mL, kPCR PLX® CMV DNA-Assay in combination with the VERSANT® kPCR molecular system, Siemens Healthcare Diagnostics, Eschborn, Germany) was documented.

### 2.4. Statistical Analysis

Data were analyzed using IBM SPSS Statistics 24 (IBM Corp., Armonk, NY, USA). Normally distributed continuous variables are shown as mean ± standard deviation (SD) and non-normally distributed continuous variables as median and 1st and 3rd quartiles (interquartile range, IQR). Absolute and relative frequencies are given for categorical variables.

Pairs of independent groups were compared using the Student’s t-test for normally distributed data, Mann–Whitney U test for non-normally distributed data, and Fisher’s exact test for categorical variables. To model the relationship between a dependent variable and one or more explanatory variables, we applied linear regression analysis.

## 3. Results

The demographic and clinical characteristics of the recipients are shown in Table 1, the outcome measures in Table 2, and the characteristics of the living donors (mean age: 52.0, range 35.7, 64.3 years, 28.6% male) who served as healthy controls are presented in Table 3. Fifty-five patients (52.9%) received a living donation. One third of the living donations were ABO-incompatible KTx (*n* = 17). Sixty-seven (64.4%) of the recipients were male. The mean age of the recipients at the time of KTx was 50.39 years (16.9–76.8 years). 

### 3.1. GDF15

**Figure 1:**
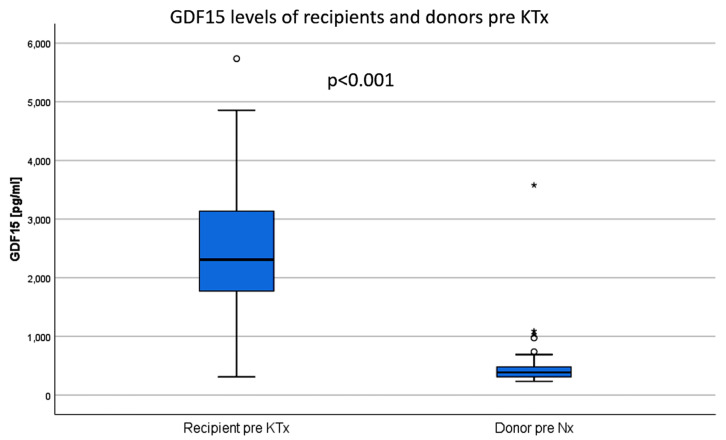
The mean GDF15 values of patients with end-stage renal disease are significantly higher than in healthy controls (living donors before donation). KTx: kidney transplantation, Nx: nephrectomy.

GDF15 levels were significantly elevated in the patients with ESRD compared to the healthy controls (LD prior to donation, 2844 (IQR 2087, 3361) pg/ml vs. 384 (IQR 307, 487) pg/ml, respectively, *p* < 0.001, Figure 1).

**Figure 2:**
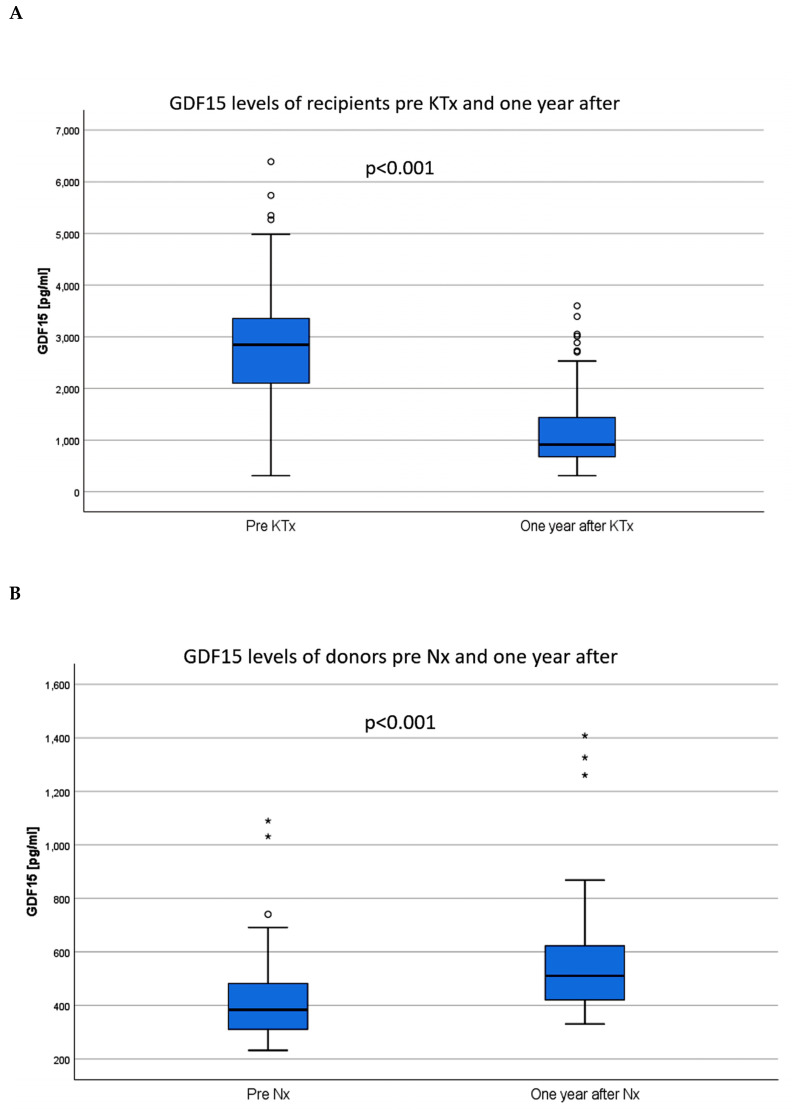

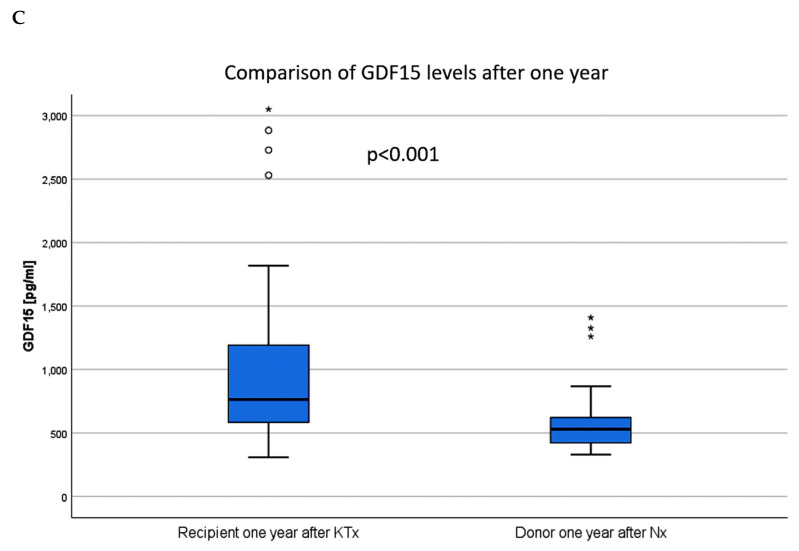
Within one year after surgery, the GDF15 values of the KTx recipients decreased (**A**), while the GDF15 values of the donors increased after nephrectomy (**B**). GDF15 levels of recipients remained significantly higher compared to the levels of donors (**C**). KTx: kidney transplantation; Nx: nephrectomy.

KTx led to a remarkable decrease of the GDF15 levels of recipients. One year after surgery, GDF15 values had decreased on average by 1,934 pg/ml to 913 (IQR 674, 1,453) pg/ml (*p* < 0.001 vs. baseline, Figure 2A). In the same period, GDF15 levels in LD increased slightly by an average of 126 pg/ml after uni-nephrectomy (Nx) (510 (IQR 420, 626) pg/ml, *p* < 0.001, Figure 2B). One year after KTx or Nx, the GDF15 value remained significantly higher in recipients than in LD (*p* = <0.001, Figure 2C).

**Figure 3:**
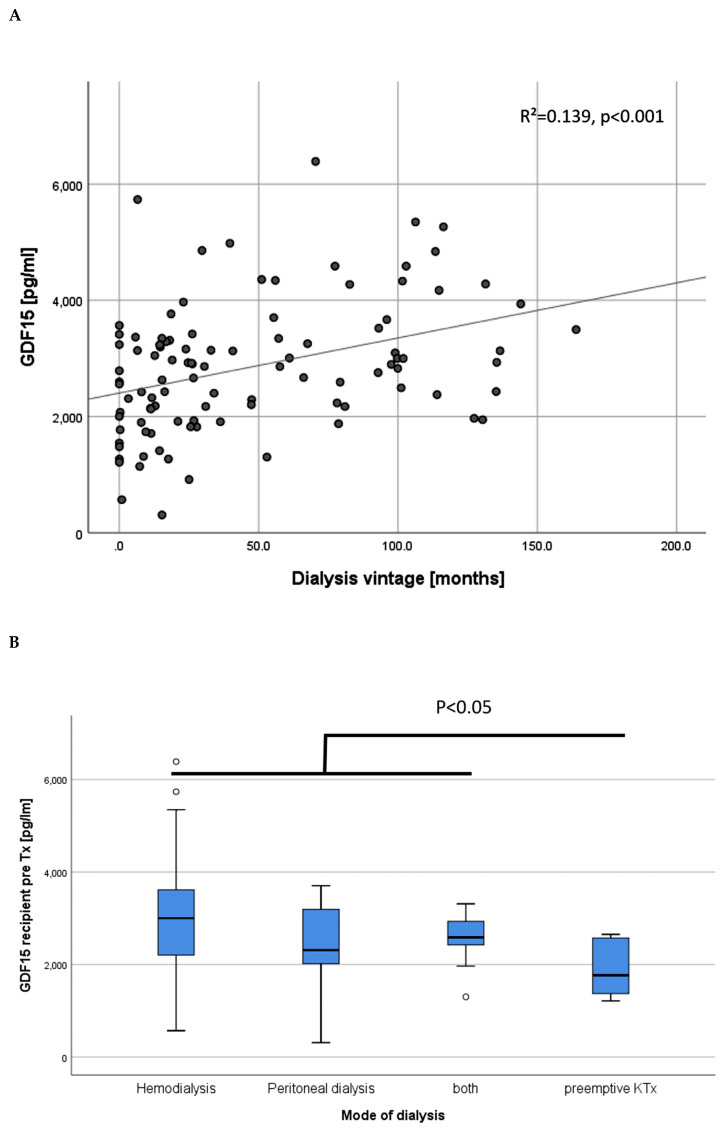

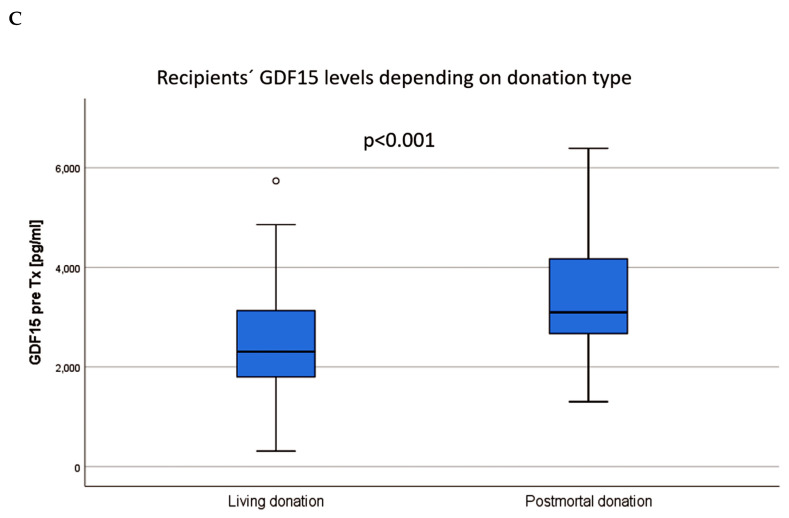
Association of GDF15 levels before KTx with the dialysis vintage (**A**). In congruence, GDF15 levels prior to KTx were significantly lower in end-stage renal disease patients without the need for dialysis treatment (**B**). The GDF15 values in recipients of a living donation were lower than in recipients of a postmortal donation (**C**).

Before KTx, the GDF15 levels of the recipients were clearly associated with the dialysis vintage (t = 4.036, *p* < 0.001, Figure 3A). Consistent with this data, patients who received preemptive KTx (*n* = 8, 7.7 %) showed the lowest GDF15 levels compared to patients treated with hemo- or peritoneal dialysis (*p* = 0.006, Figure 3B). Recipients of a LD had lower GDF15 levels than recipients of postmortal donations (2399 ± 985 pg/ml vs. 3349 ± 1046 pg//ml, *p* < 0.001, Figure 3C).

### 3.2. Renal Function

**Figure 4:**
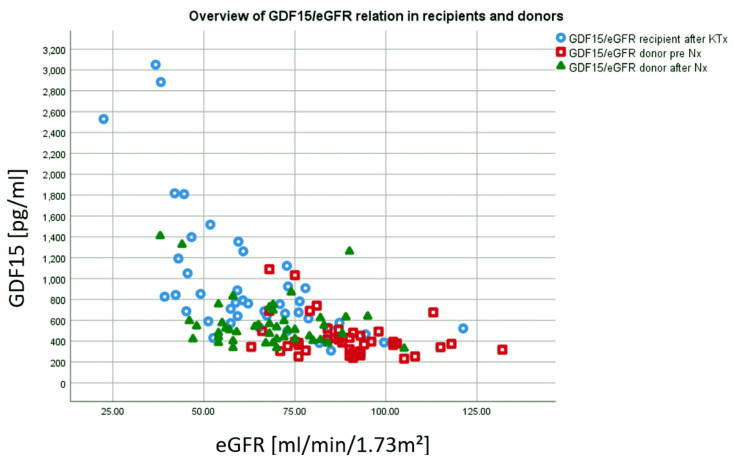
Recipients one year after KTx showed highest GDF15 values and lowest eGFR (blue). In contrast, donors´ after Nx showed lower GDF15 values and higher eGFR after Nx (green). Whereas eGFR is noticeably decreased compared to pre Nx, GDF15 values are only slightly elevated compared to pre Nx (red).

One year after KTx, the mean eGFR of the recipients was 56.8 ± 20.5 ml/min/1.3 m^2^, after two years 54.8 ± 20.5 ml/min/1.3 m^2^, after three years 53.2 ± 17.7 ml/min/1.3 m^2^ and after four years 51.3 ± 15.9 ml/min/1.3 m^2^. Donors had a mean eGFR of 88.5 ± 14.8 ml/min/1.3 m^2^ prior to kidney donation. One year after Nx, eGFR had decreased about 21.6 ml/min/1.3 m^2^ (24.4%) to an eGFR of 66.9 ± 14.5 ml/min/1.3 m^2^. An overview of the association between eGFR and GDF15 values of recipients and donors is provided in Figure 4.

**Figure 5:**
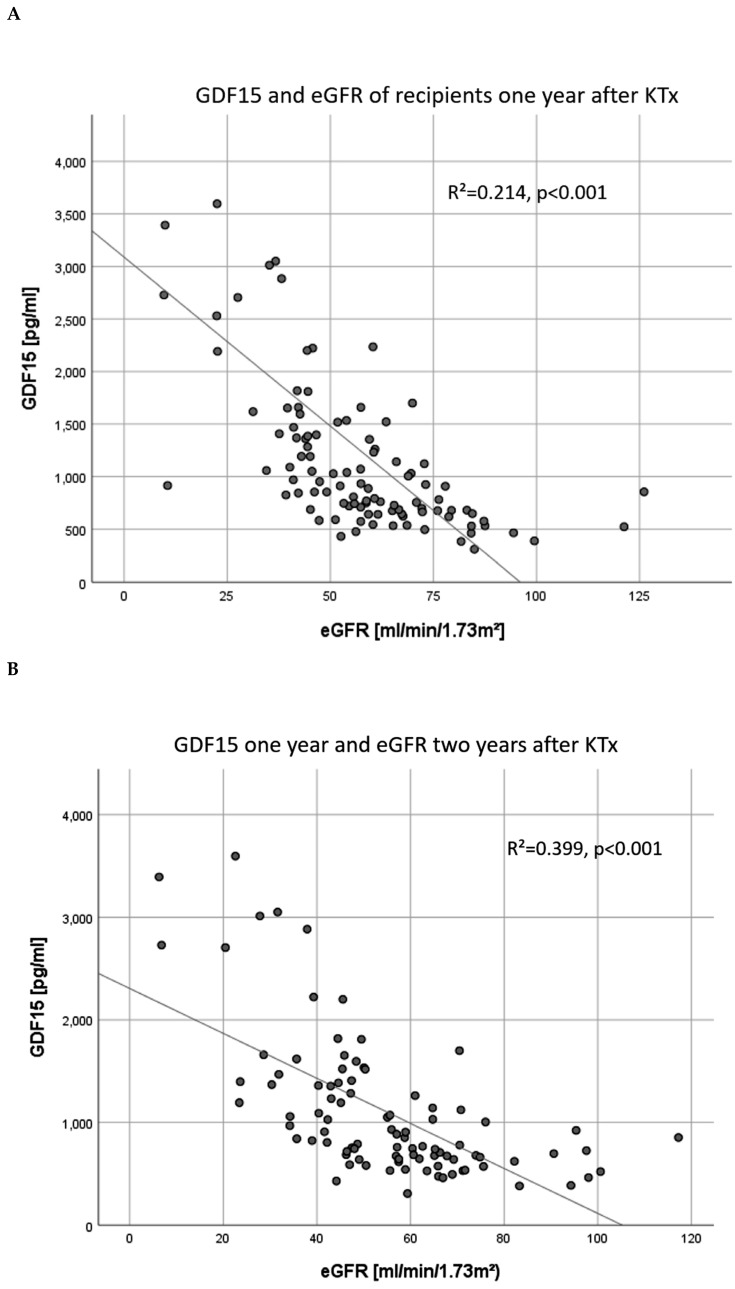

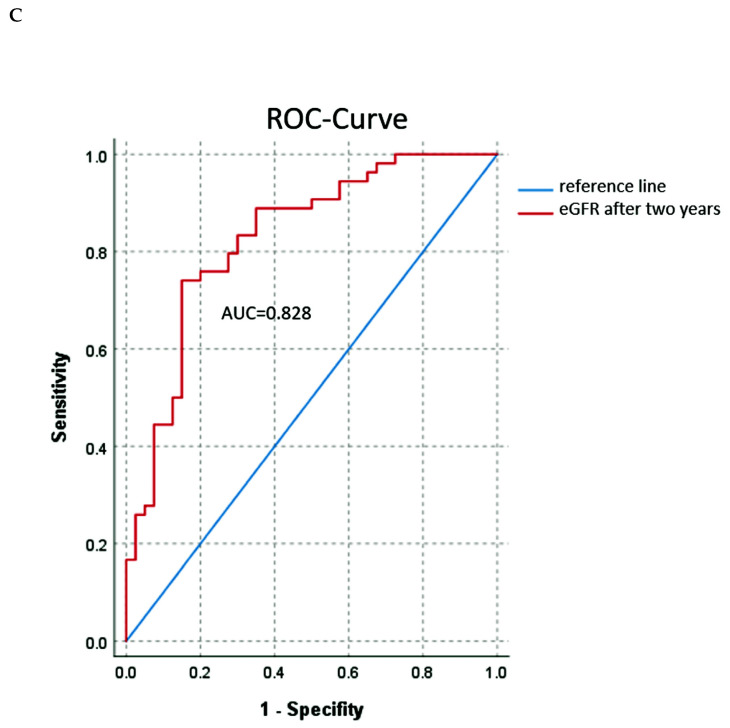
The GDF15 levels of recipients one year after KTx were significantly associated with the graft function at the same time (**A**) and also one year later (two years after KTx, (**B**)). GDF15 levels <1000 pg/ml one year after KTx predicted a better eGFR at two years after KTx (**C**). KTx: kidney transplantation.

**Figure 6:**
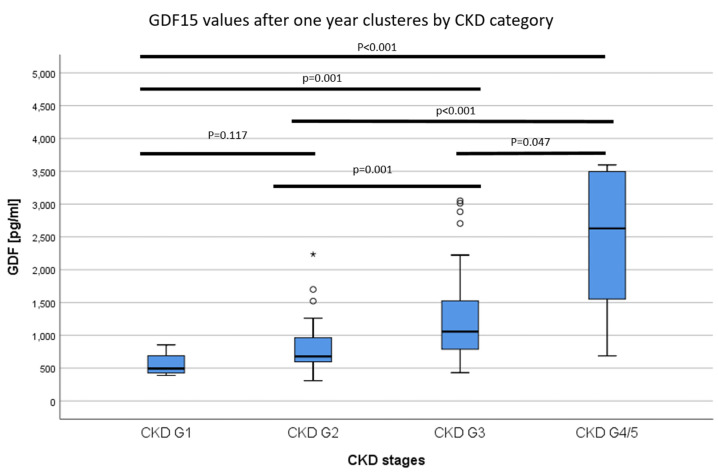
The GDF15 values of recipients one year after KTx clustered by CKD stage. GDF15 values were increased depending on the CKD category.

The GDF15 levels of recipients after one year correlated with the eGFR at 1–4 years after KTx (one year: r = −32.148, *p* = <0.001, two years: r = −21.908, *p* < 0.001, three years: r = −18.026 *p* < 0.001, four years: r = −17.897, *p* < 0.001), Figure 5A,B). One year after KTx, a GDF15 below 1000 pg/ml predicted a higher eGFR one year later (*p* = <0.001, AUC = 0.828, CI 0.743–0.914, overall model quality 0.74, Figure 5C). GDF15 values after one year were strongly associated with the CKD-stage of the recipients (Figure 6).

**Figure 7:**
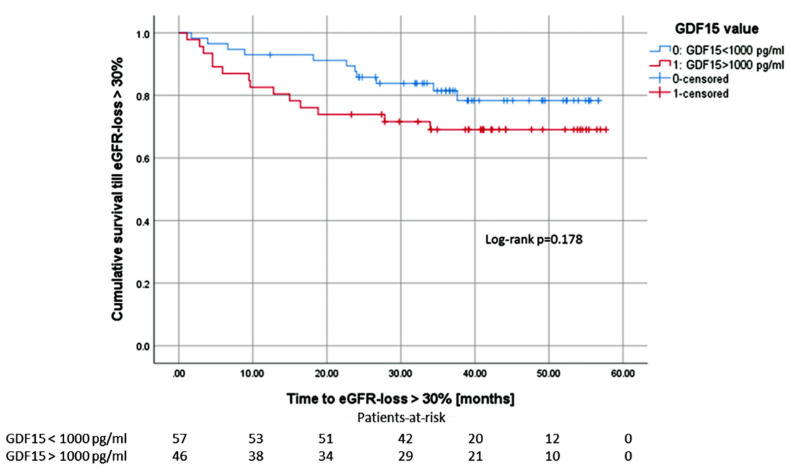
Patients with GDF15 values above 1000 pg/ml showed a higher incidence of eGFR-loss > 30% within the first 18 months after measurement compared to patients with GDF15 values below 1000 pg/ml. Taking the whole observation period into account, there is no statistically significant difference.

We analyzed the surrogate endpoint eGFR loss > 30% as the primary endpoint [18,19], starting one year after transplantation at the time of the second GDF15 measurement. Patients with GDF15 values > 1000 pg/ml (n = 46) showed a higher incidence of eGFR-loss > 30% than those with GDF15 values < 1000 pg/ml (n = 57), (eleven (23.9%) vs. four (7.0%)). Although there was a trend for worse eGFR in patients with GDF > 1000 pg/ml over the whole study period, the difference did not reach statistical significance between the groups (*p* = 0.178) (Figure 7).

In LD, GDF15 levels prior to Nx were not associated with the donors` eGFR prior to Nx (*p* = 0.208, r = −6.021) or with eGFR one year after KD (*p* = 0.289, r = −5.156). In addition, donors´ GDF15 levels one year after Nx were not associated with eGFR after Nx (*p* = 0.100, r = −3.084). 

In terms of UPCR, GDF15 at one year after KTx tended to be associated with UPCR at the same time (*p* = 0.085, r = 2.72), and when the UPCR two years after KTx was analyzed, the association became significant (*p* < 0.001, r = 1.171). However, UPCR after three years was not significantly associated with GDF15 any more (*p* = 0.118, r = 0.534).

### 3.3. Rejection Episodes

Forty-six of the patients (44.2%) were diagnosed with at least one rejection episode during the follow-up, but the incidence of at least one rejection was not associated with the GDF15 value after KTx (*p* = 0.365).

### 3.4. Tacrolimus Trough Levels and Trough Concentration/Daily Dose (C/D) Ratio

In our patient cohort, 81 patients (77.9%) received a tacrolimus-based immunosuppression one year after KTx. Neither the tacrolimus trough levels (*p* = 0.317, r = 39.178) nor the C/D ratio (*p* = 0.703, r = −27.529) were associated measurements of GDF15 values on the same day one year after KTx.

### 3.5. Cardiovascular Disease (CVD)

Of the 104 studied patients, 20 (19.2%) suffered from CVD. CVD was defined as myocardial infarction, stroke and/or symptomatic peripheral arterial disease. We evaluated, whether GDF15 values prior to KTx or one year after KTx were associated with CVD in our patient cohort. We found that patients with CVD showed significantly higher GDF15 values prior to Tx than patients without CVD (3430 ± 1049 pg/ml vs. 2708 ± 1092 pg/ml, *p* = 0.003).

The same applies to GDF15 levels one year after Tx (2120 ± 2853 pg/ml. vs. 1062 ± 650 pg/ml, *p* = 0.003).

### 3.6. Cytomegalovirus and BK Polyomavirus (BKPyV)

Since GDF15 plays a role in infection, we assessed the occurrence of cytomegalovirus (CMV) and BKPyV DNAemia in our cohort. During follow-up, 20 patients (19.2%) developed CMV DNAemia; 16 of them within the first year after KTx. The median onset time of CMV-DNAemia was 7.5 months after KTx. None of the 20 patients suffered from invasive CMV organ disease. Patients with active CMV replication within the first year showed higher GDF15 levels one year after KTx compared to patients without replication (1471 ± 785 pg/ml vs. 1212 ± 1542 pg/ml, *p* = 0.009, univariate analysis). We could not find any association between GDF15 values one year after KTx and the number of CMV copies in the peripheral blood (*p* = 0.332).

Nineteen patients (18.3%) developed BKPyV DNAemia. Patients who developed BKPyV DNAemia in the course did not show significantly different GDF15 levels after one year (1249 ± 1561 pg/ml vs. 1347 ± 731 pg/ml, *p* = 0.084, univariate analysis).

### 3.7. Overall Graft Survival

Within the follow-up period, four patients had a terminal graft failure and four patients died. Two of the patients who died lost their graft prior to death. Due to the low numbers, we refrained from further endpoint analyses.

### 3.8. Adjustment

Since CMV-DNAemia, proteinuria and rejection show a strong association with renal function [17], we adjusted these factors for the 1-year eGFR. After adjustment, CMV-DNAemia (*p* = 0.163), rejection episodes (*p* = 0.245) and proteinuria after one year (*p* = 0.968) did not show any association to GDF15. However, UPCR after two years was still significantly associated with the GDF15 level one year after KTx in recipients (*p* = 0.002).

### 3.9. Multivariable Linear Models

Multivariable linear models confirm that the GDF15 level of recipients are independently associated with eGFR one or two years after KTx (*p* < 0.001, Table 4 and Table 5) and vice versa (*p* < 0.001, Table 6). In addition to GDF15, we found that higher recipients´ age at KTx, higher donors´ age, and postmortal donation were associated with the one and two eGFR after KTx. In contrast, only eGFR was significantly associated with the GDF15 value.

## 4. Discussion

We herein show that the increased serum GDF15 values of ESRD patients decrease after KTx and then not only correlate with kidney function but are strongly predictive for kidney function one year later. In addition, we report for the first time that GDF15 increases noticeably after KD. This is important because GDF15 has been linked to the onset and progression of kidney disease. Moreover, it was suggested that it could be useful in predicting the progression of CKD, years before the clinical onset of the disease and could serve as an independent risk factor for mortality in CKD patients [3,12,20].

GDF15 is known to play a key role in various pathological conditions including CKD, metabolic disorders as well as sterile and infectious inflammation [8,9,12]. All of these factors coincide in ESRD patients receiving a kidney transplant. However, the exact pathophysiological role of GDF15 is still unknown as both protective and deleterious effects have been described [7].

Compared to healthy LD, we observed noticeably increased GDF15 levels in patients with ESRD. This is consistent with observations by others who found that CKD in all age cohorts leads to a significant increase of GDF15 [12,13,15,21]. In addition, GDF15 can also increase in response to acute kidney injuries [10,11]. Furthermore, the dialysis vintage and possibly the duration of CKD appear to play an important role in the extent of GDF15 values, as GDF15 levels before transplantation were significantly associated with the dialysis vintage in our patients. Congruently, patients with preemptive transplantation who probably experience the shortest CKD and ESRD period without need for dialysis treatment have the lowest GDF15 levels. This finding is also in line with the results of a dialysis study published by You et al. [4] and is also sufficient to explain lower GDF15 values among recipients of living donations prior to KTx as these patients are more often preemptively transplanted and have a shorter dialysis vintage on average (Table 1).

After KTx, GDF15 levels of KTx recipients significantly decreased. This is consistent with the data from Connelly et al. [13], who observed significantly lower GDF15 values among their patients one year after KTx than at baseline. Thorsteinsdottir et al. showed that kidney transplanted children had lower GDF15 values than children on dialysis [15]. This observation may be explained by the fact that KTx eliminates two triggers that increase serum GDF15 to a certain extent, namely dialysis therapy and renal insufficiency. However, patients after KTx continue to have CKD, with an eGFR of 56.8 ± 20.5 ml/min/1.3 m^2^ after one year and 54.8 ± 20.5 ml/min/1.3 m^2^ two years after transplantation (Table 2, Figure 5A and Figure 6). Therefore, our results, which show a strong association between elevated GDF15 levels and impaired allograft function one year after KTx, and the observation that GDF15 was able to predict graft function one year later, are consistent with Nair et al. who showed an increased risk for CKD progression with higher GDF15 levels in two separate, independent CKD patient cohorts (Nair et al. 2017).

In the context of acute kidney injury, the TGF-ß family member GDF15 is rapidly upregulated [11]. Further upregulation of TGF-β promotes endothelial-to-mesenchymal transition and causes fibrosis in kidneys and other organs [22]. After KTx, it is involved in the development of interstitial fibrosis and tubular atrophy (IF/TA) [23]. Recently, GDF15 was found to be associated with matrix metalloproteinases and sST2 proteins, which are molecular targets of fibrosis [24]. Moreover, it was shown that GDF15 is a TGF-ß responsive gene [25]. In mice, it is involved in podocyte pathogenesis, and in humans, GDF15 is upregulated in the glomeruli of patients with focal segmental glomerulosclerosis. Interestingly, Kim et al. proposed that GDF15 has anti-fibrotic properties in primary fibroblasts isolated from mouse kidneys with ureteral obstruction-induced fibrosis [26]. The authors showed that GDF15 is able to block the TGF-ß receptor. This type of (negative) feedback was also recently assumed by Arif et al. [25]. In consequence, GDF15 elevation in serum can indicate TGF-ß activity (in target organs). However, it remains unclear to some extend whether GDF15 is a marker or a (co-) cause of CKD. 

In terms of infections, a protective role was attributed to GDF15 [8]. In this regard, we assessed CMV-DNAemia in our KTx recipients. Interestingly, patients who developed CMV DNAemia within the first year after KTx had higher GDF15 levels after one year, but were also characterized by inferior allograft function. This is in line with CMV data from a larger cohort than our center [17]. However, after adjustment for eGFR, the association did not remain significant. Since none of the 20 patients who showed CMV replication suffered from invasive CMV organ disease, we cannot provide a statement about a conceivable difference of GDF15 values in patients with sole CMV DNAemia and those with invasive organ disease. An important limitation of the study with respect to our study design is that serum samples for GDF15 were not (necessarily) collected during acute CMV viremia. Further prospective investigations need to clarify whether GDF15 is upregulated in cases of CMV DNAemia or other viral infections due to the protective function of GDF15 against viral infections postulated by Luan et al. [8]. Ultimately, other pro-inflammatory conditions such as diabetes, previously stated to increase GDF15 levels, or rejection episodes were not associated with GDF15 in our KTx recipients [27]. However, the study cohort might be too small to detect these relations.

Our observations could have an interesting influence on the evaluation of KD since the understanding of the outcomes and risks for LD is increasingly important. We herein investigated serum GDF15 for the first time after KD and found a significant increase of the GDF15 values one year after Nx. This is important because our observations and comparisons between LD and recipients suggest that GDF15 levels exceeding about 1000 pg/ml predict a worse renal outcome in both patient groups. Moreover, this is interesting, because in contrast to CKD patients, LD typically do not develop a chronic inflammatory state or inflammation related to kidney disease or other features of CKD patients despite a decreased renal function [28,29], which is a typical inducer of the stress-responsive cytokine GDF15.

One year after LD, the eGFR decreased by about 24.4% in our donors, which corresponds to observations in other cohorts [9,28]. However, the mean eGFR still remained around 60 ml/min/1.73 m^2^. Then the question is why GDF15 is increasing or, conversely, why TGF-ß activity is increasing after LD? Interestingly, the correlation between GDF15 and eGFR in the group of LD was not preserved in opposite to recipients, hinting towards a different mechanism of GDF15 activation in LD. This is an arousing aspect since it was shown that patients with chronic fatigue syndrome, which has been linked to LD, display higher GDF15 levels than healthy controls [30,31]. Interestingly, healthy controls had GDF15 values comparable to LD values before surgery, while patients with fatigue had comparable values to LD one year after KD. It would be interesting to evaluate this finding in a prospective study.

Since it is known that the kidney itself is a source of GDF15 [10], this observation could also point towards an unfavorable TGF-ß activation in LD leading to an inferior outcome after KTx. Unfortunately, a limitation of our study is that we have no data about the (renal) GDF15 production rates and or the contribution of the kidney to the clearance of GDF15 and, of course, there are several factors such as infections or cardiovascular disease that could affect graft function and thus may nullify the feasibility of GDF15 in LD to predict graft function in recipients more sensitively. This relationship should be evaluated in a prospective study. Considering the associations of GDF15 with an unhealthy lifestyle, cardiovascular risk factors, and various age-related chronic diseases, including CKD, cardiovascular disease, cancer, and even cognitive decline, GDF15 can be considered a marker of biological age [6]. However, it is comforting to know that GDF15 decreased after KTx to the extent that most recipients (and also LD after KD) had GDF15 serum concentrations below 2,000 pg/ml; a threshold with the values above was linked to increased cardiovascular disease and heart failure rates and mortality in CKD patients [5]. This aspect is confirmed by our results, as patients with symptomatic cardiovascular disease showed mean GDF values of 2,120 pg/ml, which is twice as high as in recipients without cardiovascular disease (1,062 pg/ml).

## 5. Conclusions

In conclusion, KTx decreased the stress-responsive cytokine GDF15 serum values in recipients compared to patients with ESRD, especially to those on (hemo-)dialysis. In contrast, KD increased the GDF15 level noticeably one year after LD. After KTx and LD, GDF15 was clearly related to eGFR, not only as a biomarker of current eGFR, but also as a prognostic marker for future kidney function. A clinical consequence could be a closer monitoring and more rigorous elimination of metabolic risk factors in patients with higher GDF15 values. Further, our study should stimulate prospective studies evaluating the relationship between GDF15 of the donor and the graft as well as the role of GDF15 in LD. The question that now needs to be answered is: “Can GDF15 potentially be utilized as an additional parameter for the evaluation of LD to prevent unfavorable outcomes after KD due to early stage kidney disease not manifested by renal function or underestimated cardiovascular morbidity?” In particular, the strong response of GDF15 after KTx, and also LD, provides a rationale to prospectively assess its value in both settings.

## Figures and Tables

**Table 1:** Baseline characteristics of the transplant recipients.

	All (*n* = 104)	Living (*n* = 55)	Cadaveric (*n* = 49)	*p*-value
**Age (years, mean ± SD)**	50.39 (15.83)	42.9 (15.2)	58.8 (12.0)	<0.001 ^a^
**Male sex, *n* (%)**	67 (64.4)	34 (61.8)	33 (67.3)	0.682 ^b^
**Diagnosis of ESRD, *n* (%)**				0.766 ^b^
Hypertension	13 (12.5)	7 (12.7)	6 (12.2)	
Diabetes	3 (2.9)	0 (0)	3 (6.1)	
Polycystic kidney disease	14 (13.5)	6 (10.9)	8 (16.3)	
Obstructive Nephropathy	5 (4.8)	3 (5.4)	2 (4.1)	
Glomerulonephritis	34 (32.7)	20 (36.4)	14 (28.6)	
FSGS	7 (6.7)	5 (9.1)	2 (4.1)	
Interstitial Nephritis	3 (2.9)	1 (1.8)	2 (4.1)	
Vasculitis	3 (2.9)	2 (3.6)	1 (2)	
Other	22 (21.2)	11 (20)	11 (22.4)	
**Mode of dialysis, *n* (%)**				0.009 ^b^
HD	75 (72.1)	39 (70.9)	36 (73.5)	
PD	12 (11.6)	6 (10.9)	6 (12.2)	
Both	9 (8.7)	2 (3.6)	7 (14.3)	
Preemptive transplantation	8 (7.7)	8 (14.5)	0 (0)	
**Dialysis vintage (months, median (1^st^, 3^rd^ quartile))**	26.8 (11.41, 80.95)	12.3 (0.75, 25.6)	79.2 (38.0, 104.6)	<0.001 ^a^
**European senior program (ESP)**	17 (16.3)	0 (0)	17 (34.7)	<0.001 ^b^
**≥1 prior kidney transplant, *n* (%)**	14 (13.4)	8 (14.5)	6 (12.2)	0.882 ^b^
**HLA mismatch, *n* (%)**				0.088 ^a^
0–3	62 (59.6)	32 (58.2)	30 (61.2)	
4–6	41 (39.4)	22 (40.0)	19 (38.8)	
**Current PRA, *n* (%)**				0.419 ^a^
0–20%	76 (73.1)	41 (74.5)	35 (69.4)	
>20%	28 (26.9)	14 (25.5)	14 (30.6)	
**Induction therapy, *n* (%)**				<0.001 ^b^
Basiliximab	78 (75)	37 (67.3)	41 (83.7)	
Thymoglobulin	6 (5.8)	0 (0)	6 (12.2)	
Basiliximab + Thymoglobulin	1 (0.9)	0 (0)	1 (2.0)	
Rituximab + Thymoglobulin	1 (0.9)	1 (1.8)	0 (0)	
Rituximab	16 (15.4)	16 (29.2)	0 (0)	
Eculizumab + Basiliximab	2 (2.9)	1 (1.8)	1 (2.0)	
**Cold ischemia time (hours, mean ± SD)**	6.03 (4.4)	2.52 (0.55)	9.9 (3.4)	<0.001 ^a^
**Warm ischemia time (min, mean ± SD)**	34.6 (7.6)	34 (7.9)	35.4 (7.3)	0.200 ^a^
**AB0i**	17 (16.3)	17 (30.9)	0 (0)	<0.001 ^b^

Demographic characteristics of the study population. Results are presented as mean ± standard deviation (SD) or median and 1st and 3rd quartile, respectively, or as absolute and relative frequencies. ESRD: end-stage renal disease; FSGS: focal segmental glomerulosclerosis; ESP: European Senior Program; HLA: human leukocyte antigen; PRA: panel reactive antibodies. ^a^ Mann–Whitney U test; ^b^ Fisher’s exact test; ^c^ Kruskal–Wallis test.

**Table 2:** Outcomes of the recipients.

	All (*n* = 104)	Living (*n* = 55)	Cadaveric (*n* = 49)	*p*-value
**GDF15 pre KTx (median (1^st^, 3^rd^ quartile)**	2,844 (2087, 3,361)	2,299 (1763, 3,137)	3,096 (2,631, 4,223)	<0.001 ^a^
**GDF15 day 365 median (1^st^, 3^rd^ quartile)**	913 (674, 1,453)	775 (611, 1,139)	1,141 (747, 1,636)	<0.001 ^a^
**eGFR day 365 (ml/min/1.73m², mean ± SD)**	61.1 (18.6)	64.6 (16.9)	57.5 (19.9)	0.036 ^a^
**eGFR day 720 (ml/min/1.73m², mean ± SD)**	59.9 (19.0)	62.5 (15.5)	57.2 (22.0)	0.107 ^a^
**eGFR day 1080 (ml/min/1.73m², mean ± SD)**	56.0 (18.1)	58.5 (16.2)	53.3 (19.8)	0.068 ^a^
**eGFR day 1440 (ml/min/1.73m², mean ± SD)**	52.6 (15.4)	54.7 (13.9)	50.4 (16.7)	0.207 ^a^
**UPCR day 365 (mg/g crea, mean ± SD)**	135 (114)	131 (82)	140 (141)	0.832
**UPCR day 720 (mg/g crea, mean ± SD)**	160 (214)	131 (106)	191 (285)	0.344 ^a^
**UPCR day 1080 (mg/g crea, mean ± SD)**	165 (208)	167 (247)	163 (163)	0.594 ^a^
**UPCR day 1440 (mg/g crea, mean ± SD)**	169 (205)	175 (244)	163 (159)	0.978 ^a^
**eGFR-loss > 30% from year one, *n* (%)**	25 (24.0)	15 (27.3)	10 (20.4)	0.496
**Rejection, *n* (%)**	46 (44.2)	28 (50.9)	18 (36.7)	0.170 ^b^
Antibody-mediated rejection	14 (13.5)	10 (18.2)	4 (8.2)	
T-cellular rejection	4 (3.8)	3 (5.5)	1 (2.0)	
Combined rejection	4 (3.8)	2 (3.6)	2 (4.1)	
T-cellular borderline rejection	23 (22.1)	12 (21.8)	11 (22.4)	
**CMV viremia, *n* (%)**	20 (19.6)	4 (7.3)	16 (32.7)	0.001 ^b^
**BKPyV viremia, *n* (%)**	19 (18.3)	6 (10.9)	13 (26.5)	0.043 ^b^
**NODAT, *n* (%)**	19 (18.3)	6 (10.9)	13 (26.5)	0.046 ^b^

eGFR: estimated glomerula filtration rate, calculated by CKD-EPI formula; UPCR: urine protein creatinine ratio; CMV: Cytomegalovirus; BKPyV: BK-Polyomavirus; NODAT: New onset diabetes after transplantation. ^a^ Mann-Whitney U test; ^b^ Fisher’s exact test.

**Table 3:** Kidney donors: Baseline characteristics and laboratory results.

Variables Baseline	
Patients (n)	54
Age at donation [years] (min, max)	52.0 (35.7, 64.3)
Sex male (%)	18 (28.6%)
BMI (kg/m^2^± SD)	24.0 (6.6)
**Variables Outcome**	
GDF15 pre donation (pg/mL, median (1^st^, 3^rd^ quartile))	384 (307, 487)
GDF15 day 365 (pg/mL, median (1^st^, 3^rd^ quartile))	510 (420, 626) ^a^
eGFR before donation [mL/min/1.73m^2^]	95.9 (17.0)
eGFR day 365 (mL/min/1.73m^2^ ± SD)	69.0 (15.8) ^a^
UPCR before donation (mg/g crea± SD)	79.5 (62.9)
UPCR day 365 (mg/g crea ± SD)	86.1 (41.2) ^b^

BMI: body mass index; eGFR: estimated glomerula filtration rate, calculated by CKD-EPI formula; UPCR: urine protein creatinine ratio; ^a^ *p* ≤ 0.001 vs. pre donation; ^b^ *p* = 0.306 vs. pre donation.

**Table 4:** Multivariable linear regression model for eGFR one year after KTx.

Variable	Regression-coefficient B	95% CI	*p* value
GDF15 365 days	−0.005	−0.008–−0.003	<0.001
Patient age at KTx	0.563	−0.871–−0.255	<0.001
No previous KTx	−3.072	−13.819–7.674	0.571
CMV mm D-/R-	7.076	−1.880–16.031	0.120
CMV mm D+/R-	5.799	−6.423–18.021	0.348
CMV mm D-/R+	−0.254	−9.274–8.766	0.955
CMV mm D+/R+	0 ^a^	.	.
Postmortal KTx	−19.184	−34.228–−4.140	0.013
Donor age	−0.326	−0.697–0.046	0.085
Dialysis vintage	−0.092	−0.208–0.025	0.123
Cold ischemia time	−1.012	−2.802–0.778	0.264
Warm ischemia time	0.176	−0.279–0.631	0.444
PRA	0.006	−0.114–0.126	0.921
HLA-mismatches	1.758	−0.880–4.396	0.189

^a^ Parameter 0, because redundant; KTx: Kidney transplantation; CMV: Cytomegalovirus; mm: Mismatch; D/R: Donor/Recipient; PRA: Panel reactive antibodies; HLA: Human leukocyte antigen.

**Table 5:** Multivariable linear regression model for eGFR two years after KTx.

Variable	Regression-Coefficient B	95% CI	*p* Value
GDF15 365 days	−0.015	−0.021–−0.010	<0.001
Patient age at KTx	−0.372	−0.670–−0.075	0.015
No previous KTx	5.135	−5.061–15.330	0.319
CMV mm D-/R-	2.095	−6.371–10.562	0.623
CMV mm D+/R-	3.239	−9.026–15.504	0.600
CMV mm D-/R+	−1.445	−9.849–6.960	0.733
CMV mm D+/R+	0 ^a^	.	.
Postmortal KTx	−22.898	−37.328–−8.467	0.002
Donor age	−0.544	−0.898–−0.190	0.003
Time of dialysis	−0.101	−0.213–0.011	0.076
Cold ischemia time	0.969	−2.659–0.720	0.257
Warm ischemia time	0.143	−0.293–0.579	0.516
PRA	0.041	−0.077–0.160	0.491
HLA-mismatches	1.747	−0.814–4.308	0.178

^a^ Parameter 0, because redundant; KTx: Kidney transplantation; CMV: Cytomegalovirus; mm: Mismatch; D/R: Donor/Recipient; PRA: Panel reactive antibodies; HLA: Human leukocyte antigen.

**Table 6:** Multivariable linear regression model for GDF15 one year after KTx.

	Regression-coefficient B	95% CI	*p* value
eGFR 365 days	−36.979	−53.547–−20.411	<0.001
Patient age at KTx	−6.908	−34.639–20.823	0.622
No previous KTx	−359.404	−2524.879–1806.070	0.742
No CMV viremia	192.050	−608.566–992.665	0.635
Postmortal KTx	−649.883	−2037.429–737.663	0.354
Donor age	−18.420	−48.669–11.829	0.229
Time of dialysis	−8.644	−18.358–1.071	0.080
Cold ischemia time	0.255	−153.523–154.034	0.997
Warm ischemia time	3.154	−34.709–41.017	0.869
PRA	−0.106	−10.118–9.905	0.983
HLA-mismatches	187.076	−34.100–408.253	0.096

eGFR: estimated glomerular filtration rate; KTx: Kidney transplantation; CMV: Cytomegalovirus; mm: Mismatch; D/R: Donor/Recipient; PRA: Panel reactive antibodies; HLA: Human leukocyte antigen.
